# Extending the theory of planned behavior model to explain people’s behavioral intentions to follow China’s AI generated content law

**DOI:** 10.1186/s40359-024-01824-4

**Published:** 2024-06-26

**Authors:** Jie-Chun Li, Yi Lin, Yi-Chun Yang

**Affiliations:** 1 School of Marxism, Zhuhai City Polytechnic, Zhuhai, China; 2 School of Public Administration, Jilin University, Jilin, China; 3 Faculty of Business and Management, Beijing Normal University-Hong Kong Baptist University, United International College (UIC), Zhuhai, China

**Keywords:** Theory of planned behavior (TPB), Behavioral intentions, Moral obligation, AI generated content law

## Abstract

AI Generated Content Law was extensively promoted in 2023; hence, it is crucial to uncover factors influencing people’s behavioral intentions to comply with the AI Generated Content Law. This study extends the theory of planned behavior to explore the factors influencing people to follow AI Generated Content Law in China. In addition to the factors in TPB model, such as one’s attitudinal factors, normative factors, and perceived behavioral control, we add another factor-moral obligation to extend the theory of planned behavior model. We used convenient sampling and there were 712 effective samples. Using the statistical software Amos17.0, the result shows that attitude, subjective norms, perceived behavioral control and moral obligation all have positive effects on intentions to follow AI Generated Content Law.

## Introduction

In recent years, digital advances have brought us a wide variety of systems with vast capabilities, such as automation, data analytics, artificial intelligence (AI) and so on [1]. Despite the benefits from digital technologies, digital advances can be at risk of application for unintended purposes [2].

In this context, the Chinese government pays attention to the seriousness of related problems and formulates a series of AI Generated Content Law to reduce the related problems. The document issued by the Chinese government emphasizes that the provision and use of generative artificial intelligence services should abide by laws and administrative regulations, and respect social morality and ethics [3]. In 2023 the AI Generated Content Law was extensively promoted; hence, it is crucial to uncover factors influencing people’s behavioral intentions to comply with the AI Generated Content Law.

With the advancement of AI, related studies have been steadily increasing, primarily concentrating on the applications of AI [4], few research explore people’s formations of beliefs and their influences on intentions to follow related regulations [2]. To address this gap, this study adopted the theory of planned behavior (TPB) to explore factors influencing people’s behavioral intentions. The assumption of TPB model is that some conscious factors may influence the formation of intentions to perform a specific behavior, such as attitudes, subjective norms, and perceived behavioral control [5]. In TPB theory, attitude is an important psychological emotion that can predict a person’s behavioral intention [6]. Subjective norms are proven to have significant effects on people’s intentions [7]. And perceived behavioral control is a determinant of people’s behavioral intention [8]. Because this model is extensively applied to explain a wide range of behavioral intentions, this study uses the model to explore the factors influencing people to follow AI Generated Content Law in China.

In addition to the factors in TPB model, such as one’s attitudinal factors, normative factors, and perceived behavioral control, we add another factor-moral obligation to extend the theory of planned behavior model. Moral obligation refers to the extent to which an individual feels a sense of responsibility to act (or not) morally (or immorally) when conduct an ethical situation [9]. It is suggested that in parallel with attitudes, subjective norms, and perceptions of behavioral control, adding an individual’s moral obligations as a predictor of behavior has significantly improved the prediction of intentions [9, 10].

**Fig. 1:**
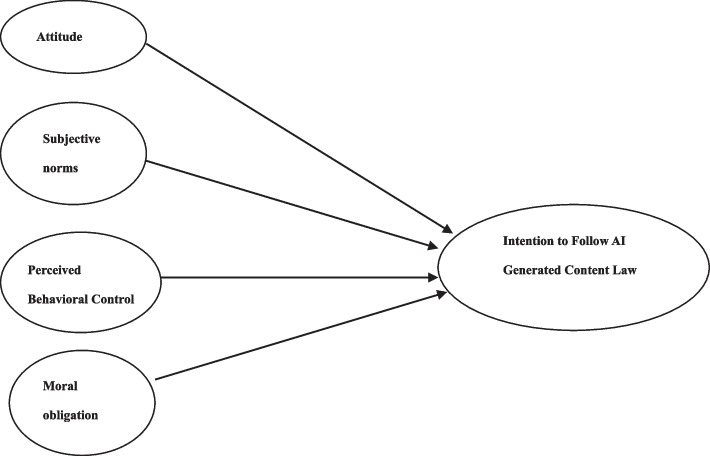
Conceptual model

AI is being extensively used to automate tasks, improve efficiency, and make better decisions [1]. However, the use of AI also raises a number of ethical concerns. There is a huge demand in people’s intensions to follow the related law. It is essential to ensure that people can comply with legal and ethical standards to avoid unintended consequences. The research question is to uncover what factors influencing people’s behavioral intentions to comply with the AI Generated Content Law. The aim is to explain people’s intentions to engage in the AI Generated Content Law based on the TPB model in China. In addition, we want to explore whether or not people’s moral obligation included in the TPB model can be a predictor to determine people’s behavioral intentions. Based on the description of research background, we propose the following conceptual model (Fig. 1):

### Relevant literature and hypotheses development

According to TPB, one’s attitudinal factors, normative factors, and perceived behavioral control determine his/her behavioral intention [5]. Understanding individual behavioral pattern is one of the key factors in formulating successful related policies. Moreover, some research regard moral norm as additional independent predictor of intention besides attitude, subjective norm, and perceived behavioral control [9]. Moral obligation is proven to significantly improve the prediction of intentions to act in a moral manner [9]. Therefore, this research used the extended theory of planned behavior model to analyze the impacts of attitude, subjective norms and perceived behavioral control on the intention to follow AI Generated Content Law.

#### The theory of planned behavior model

The theory of planned behavior, first proposed by Ajzen, assumes that an individual’s intention to engage in behavior is influenced by attitudes, subjective norms, and perceived behavioral control [11–13]. The TPB model is based on the assumption that some conscious reasoning is involved in the formation of intentions to perform specific behaviors [14–16]. TPB was applied to support the consideration of psychological factors in self-controlled and socially influenced decision-making [15, 17, 18]. The TPB is based on the idea that people generally take into account implications of their behaviors before they decide whether to engage in certain behaviors [11, 19]. Recently, this model has been widely used to explore the intention of people’s behavioral intentions [20, 21].

In TPB model, subjective norms are crucial factors in social science studies due to their strong influences on individual behavior [22–24]. Ostrom [25] proposed that subjective norms are the acceptable behaviors within general groups of certain communities [26, 27]. That is, subjective norms are important determinants of perceived intentions in social pressure to perform or not perform certain behaviors [7, 28].

Attitude refers to the degree that a person has a favorable or unfavorable evaluation toward a specific behavior developed from beliefs about some object [29, 30]). Attitude can be defined as a psychological tendency expressed by evaluating a particular entity with some degree of favor or dislike [29, 30]. Attitude can be positive or negative, and it may create positive or negative expectations of a particular outcome [31]. That is, Attitude can be viewed as a rational-choice-based evaluation of the consequences of a behavior [32].

Perceived behavioral control refers to the ease or difficulty that an individual experiences when aiming to exhibit a certain behavior [33], which has a positive effect on intentions [34]. For example, Wang [35] found that perceived behavioral control has a positive and significant effect on intention to recycle. Yadav and Pathak [36] found that perceived behavioral control was the most significant predictor for predicting intention to buy organic food.

In addition to personal desire and attempt, it also includes time, money, skills, opportunities, abilities, resources, and other uncontrollable factors of an individual that relate to the control of individual behavior [31].

Behavioral intention refers to an individual’s specific behavior and the degree of actual action [31], which can determine people’s actual behavior. TPB explained that behavioral intention is influenced by three independent variables, namely attitude, subjective norms, and perceived behavioral control [33]. However, there is a possibility that an individual may change their intention to perform actual behavior because of the existence of obstacles [29, 30].

The TPB model has been extensively used in a wide variety of behavior; nevertheless, the theoretical model has been criticized for neglecting moral consideration [37]. A person’s belief about exhibiting a specific behavior is often associated with moral norms. His belief in moral rectitude is related to moral norms while performing a specific behavior [37]. This suggests that an individual’s feeling of moral obligation to perform a certain behavior should be considered [38]. Moral obligation has significantly improved the prediction of intentions to act in a moral manner [9]. For example, Chen and Tung [39] found that a person’s moral norms can predict his/her behavioral intention to recycle waste. Chen and Tung [40] proved that a person’s perceived moral obligation can determine his/her intention to visit green hotels. It is assumed that the extended TPB model including a person’s moral obligation is better than that of the original one [41]. The extended TPB model with moral obligation can increase the explanatory power of people’s intentions to follow AI Generated Content Law.

#### Research hypothesis

In this TPB theory, attitude toward behavior positively influences intention which means positive judgment toward behavior drives intention to perform behavior [6]. Attitude is an important psychological emotion that can predict persons’ behavioral intentions [6]. The effect of attitude is significant and positively related to people’s behaviors [42]. The relationship suggests that when an individual has a positive attitude towards AI Generated Content Law, it may make people show intentions to follow the related regulations [15]. Hence, this research proposes the following hypothesis:

- Hypothesis 1. *Attitude towards AI Generated Content Law is positively affecting behavioral intention*.

Subjective norms can be explained as the strengths of normative beliefs and the motivations to follow the beliefs, which means that an individual is affected by social pressure which may dispose him to engage in a behavior [33]. Prior research suggested that subjective norms have significant effects on people’s intentions [7]. Due to a significant positive relationship between subjective norms and intention, the research hypothesis is proposed:

- Hypothesis 2. *Subjective norms are positively affecting behavioral intention*.

Perceived behavioral control refers to the perceived ease or difficulty of performing the behavior, and the amount of control one has over the attained the behavior’s goals [11]. Prior research has identified that perceived behavioral control is an important component influencing people’s behaviors [8]. The more people are able to have control over the opportunities and resources they have to perform a specific behavior, the more likely they will engage in such a behavior [43]. Therefore, this research proposes the following hypothesis.

- Hypothesis 3. *Perceived behavioral control is positively affecting behavioral intention*.

Perceived moral obligation, a personal internal state construct, is related to the extent to which an individual feels a sense of responsibility to act (or not) morally (or immorally) when faced with an ethical situation [9, 44]. Some research viewed moral norm as an additional independent predictor of intention in addition to attitude, subjective norm, and perceived behavioral control [42]. Prior research indicated that a person’s moral obligation is a determinant of people’s behavioral intentions [9]. Hence, the hypothesis is proposed.

- Hypothesis 4. * Moral obligation is positively affecting behavioral intention*.

### Methodology

#### Sample and procedures

The population is those who having the experiences of following AI related law in China. This study gathered data from Guangzhou of China, we used convenient sampling and online questionnaire survey to gather data. The targets are Guangzhou’s people having the experiences of following AI related law before in China. To confirm the eligibility of the participants, we confirm that the participants have the prior experiences, then we sent them the questionnaire via wechat. Data were collected online using wechat platform, the link to the survey was shared via wechat. Data collection was conducted between October 2023 to December 2023. The chosen 800 targets are those who have the experiences of following China’s AI Generated Content Law. All measurement items are adopted from prior studies, which were previously validated. Finally there were 712 effective samples, suggesting a response rate of 89%.

Among the 712 respondents, 54.6% were men and 45.4% were women. In addition, approximately 50% of the respondents were aged between 31 and 40 years. Education levels were fairly high, with over 69% having been educated at college level. The majority (54%) of the respondents was not married. Among the respondents, 48% reported a personal monthly income of between CNY$8,000 to CNY$10,000.

#### Measures

The measurement used in this questionnaire was based on a five-point Likert scale ranging from “strongly disagree” to “strongly agree”. The measures to assess each variable were collected and adapted from prior investigations. Attitude was measured by five items used in the TPB literature [31]. Subjective norms were measured using four items based on prior studies on the extended TPB [45]. Perceived behavioral control was measured by three items used in previous literature [31]. To measure moral obligation, two items were used to measure a person’s moral obligation to follow AI Generated Content Law [9]. Intention to follow AI Generated Content Law included five items in the extended TPB study, which were modified to suit the content of this study [45] (Table 1).

**Table 1:** Descriptive statistics of questionnaire items

Construct	Questionnaire Items
Attitude	I think following AI Generated Content Law in daily life ATT1: Is wise ATT2: Is a good idea ATT3: Is positive behavior ATT4: Is worthwhile ATT5: Is beneficial
Subjective norms	SN1: Most people who are important to me think we should carry out following AI Generated Content Law SN2: People in my organization (school, university, company) want to carry out following AI Generated Content Law SN3: Most people who are important to me think following AI Generated Content Law is a good thing SN4: Most people who I am familiar with think it is useful to carry out following AI Generated Content Law
Perceived behavioral control	PBC1: It’s easy for me to carry out following AI Generated Content Law PBC2: Carrying out following AI Generated Content Law is a decision that is only up to me PBC3: I am confident that if I want, I can carry out following AI Generated Content Law
Moral obligation	MB1: I have a moral obligation to follow AI Generated Content Law MB2: I have an obligation to future generations to follow AI Generated Content Law
Intention to follow AI Generated Content Law	I1: I am willing to follow AI Generated Content Law I2: I will suggest others to follow AI Generated Content Law I3: I will spend little time following AI Generated Content Law I4: I think it is easy to follow AI Generated Content Law I5: I definitely want to follow AI Generated Content Law in the near future

## Result

### Reliability and validity

In term of reliability, all the constructs range is higher than the standard of 0.6 [46], suggesting suitable reliability. The discriminant validity between the constructs was assessed using the method that factor loading appears higher than 0.5 [47], which indicates the extent to which the ratings of items depend on the latent variable (Table 2). Evidence of convergent and discriminant validity is supported when the average variance extracted (AVE) is greater than 0.5 for each construct and the square root of AVE is significantly higher than the correlations between constructs [47]. Overall, all the constructs in the study performed adequate reliability and validity.

**Table 2:** Descriptive statistics and correlations among indicator variables

Variables	M	SD	AVE	(1)	(2)	(3)	(4)	(5)
Attitude (1)	4.02	0.96	0.66	**0.789**				
Subjective norms (2)	4.05	0.87	0.64	0.418*	**0.802**			
Perceived Behavioral Control (3)	4.07	0.89	0.61	0.385*	0.403*	**0.811**		
(4) Moral obligation	4.03	1.06	0.59	0.349*	0.347*	0.376*	**0.796**	
Intention to Follow AI Generated Content Law (5)	4.03	0.94	0.61	0.408*	0.368*	0.417*	0.391*	**0.792**

*p < 0.05

### Data analysis

**Fig. 2:**
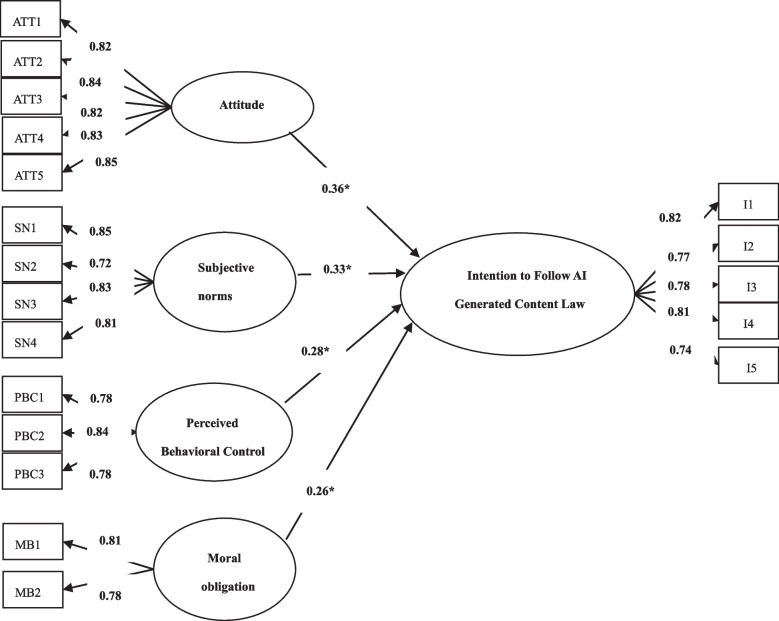
Research model

AMOS 21 was used to perform structural equation modeling to test the proposed hypotheses. Figure 2 shows a good fit (incremental fit index = 0.93; Tucker–Lewis index = 0.92; comparative fit index = 0.92; root mean square error of approximation = 0.07; chi square/degree of freedom = 2.21; Goodness of fit index: 0.93) [47]. Table 3 presents the final results for the overall sample.

### Hypothesis testing

Figure 2 indicated that attitude, subjective norms, perceived behavioral control and moral obligation all exert positive effects on intention to follow AI Generated Content Law.

As observed in Table 3, the result indicated that attitude is positively associated with intention to follow AI Generated Content Law, suggesting that H1was supported. With regard to hypothesis 2, the result proved that subjective norms positively influence intention to follow AI Generated Content Law. Hypothesis 3 is proven that perceived behavioral control positively influenced intention to follow AI Generated Content Law. Hypothesis 4 was also supported that moral obligation has a positive effect on intention to follow AI Generated Content Law. The results suggested that attitude, subjective norms, perceived behavioral control and moral obligation have positive influences on intention to follow AI Generated Content Law. This indicated that attitude, subjective norms, perceived behavioral control and moral obligation are crucial predictors of intention to follow AI Generated Content Law.

**Table 3:** Results of hypotheses and model statistics

	Path coefficient	t-Value	Results
Attitude → Intention to follow AI Generated Content Law	0.36	5.09*	Supported
Subjective norms → Intention to follow AI Generated Content Law	0.33	4.52*	Supported
Perceived behavioral control → Intention to follow AI Generated Content Law	0.28	3.72*	Supported
Moral obligation → Intention to follow AI Generated Content Law	0.26	3.61*	Supported

*p < 0.05

## Discussion and conclusion

### Discussion

To understand the policy effect of the AI Generated Content regulation, this study extends the theory of planned behavior to explore the factors influencing people to follow AI Generated Content Law in China. From the results of the empirical analysis, the key findings of this study are as follows:

Overall, the results fully confirmed the proposed hypotheses that behavior intention to follow AI Generated Content Law is determined by attitude, subjective norms, perceived behavioral control and moral obligation. The empirical findings are congruent with the conclusions in other recent literature [48]. Specifically, subjective norms have positive effects on intention to perform specific behavior, which are similar to the results of prior research [7]. This suggests that people would consider the opinions of their familiars, such as relatives and friends, while following AI Generated Content Law. This is related to the collective consciousness emphasized in daily life, where individual decisions on behavioral intentions tend to be congruent with the collective. Perceived behavioral control has a positive influence on behavioral intention, which is consistent with the prior research that perceived behavioral control is a crucial component influencing people’s behavior [8]. Attitude has a positive effect on intention to use, indicating that the more positive people’s attitudes toward the law are, the greater their intentions to follow the law. The results of the study verify that when a person has a more positive attitude toward the AI Generated Content Law, people will have positive behavioral intentions to follow the law. Finally, the effect of moral obligation is proved to have a positive effect on behavioral intention, the empirical finding is consistent with the results of Brody et al. [3] that moral obligation plays an important role in predicting one’s intention to engage in a specific behavior.

The TPB theoretical model is used to explain a wide range of intentions and behaviors in different research areas [49, 50]. The results of this research verify that one’s attitude, subjective norms and perceived behavioral control are contributors to people’s intentions to follow the AI Generated Content Law. Attitude, subjective norms and perceived behavioral control are crucial factors influencing people to follow the AI related law. In addition, when an individual has a strong moral obligation, he/she is more likely to have the intention to follow the law. Moral obligation was integrated into the original TPB model, and an extended TPB model was established to investigate the factors influencing the intention to follow AI Generated Content Law. It is found that the research model can well explain people’s intentions to comply with the law. Attitude, subjective norm, perceived behavioral control and moral obligation all have positive effects on the intention to follow the law. This research enhanced validation by examining the factors of TPB model in explaining one’s intention to perform AI-friendly behavior. In addition, we advance the knowledge by verifying that one’s moral obligation plays an important role in the extended TPB model. One’s perceived moral obligation in the extended TPB model is a predictor in predicting one’s intention to follow AI Generated Content Law. This implies that, to enhance one’s intention to follow AI Generated Content Law, both governmental and nongovernmental policy makers could be more effective by paying more attention to ways to place people under greater moral obligation to related issues. The applicability of moral obligation to the domain of people’s conscious decision making has been verified and the effect of moral obligation should not be ignored for future research designs. Based on the findings of this empirical study, the extended TPB model seems to be an ideal theoretical model for predicting people’s intentions to engage in AI related behaviors. The conclusion of the study provides management suggestions for the government to publicize the related law effectively.

This research enriches the existing research in some ways. First, few studies explored factors influencing people’s intentions to follow AI Generated Content Law, this study applied the extended theory of planned behavior model to analyze the impacts of attitude, subjective norms and perceived behavioral control on the intention to follow AI Generated Content Law. Second, we add people’s moral obligation in the TPB model as a predictor, it enables us to understand more about people’s intentions to engage in AI-regulated law.

### Theoretical and practical implications

This article has made two theoretical contributions to the researchers in related area. First of all, the findings suggest that TPB model is suitable for the study of behavioral intention towards AI related issue. This research broadens the application field for the TPB model. Second, in this study, we add moral obligation to form an extended TPB model. The study enhanced validation by examining the extended TPB model which includes one’s moral obligation to understand one’s intention to follow AI Generated Content Law in China, we verify that one’s moral obligation is also an important role in the extended TPB model.

In addition to theoretical implications, this study provides the lawyers with recommendations for effectively managing the implementation of AI Generated Content Law. The implementation of law needs to be vigorously publicized by the lawyers. Because following the law is a behavior asked of each citizen in society, publicity by the lawyers should be spread throughout society as a whole. In the beginning of the implementation of related law, the lawyers should increase publicity efforts to help people accept this new law ideology and form new habits. In addition, lawyers should make people more familiar with the knowledge, so they can effectively follow the related law. Lawyers can also try to use some tools to enhance people’s attitude toward the related law. Finally, Law makers can pay more attention to ways to place people under greater moral obligation to their AI activities. The conclusion of the study provides regulatory suggestions for the law makers to publicize the related law effectively.

### Limitations and future research

The current research poses certain limitations that lead to other avenues for future study. First, the study was based on data collected from participants in China, and the samples may have specific representativeness and particularity. The sample may not be representative of the China’s population as a whole. Future research can test the generalization of finding in other contexts. Second, causality could not be determined, future research, preferably using a longitudinal design to provide evidence of the causal linkages proposed in the model, and the design should be in very specific conditions which meet to determine causality. Finally, it is possible that the proposed model may not capture all of constructs that influence people’s behavioral intentions to follow AI Generated Content Law, future studies may work on potential factors that may influence the behavioral intentions.

## Data Availability

The datasets used and analysed during the current study available from the corresponding author on reasonable request.
